# A Dataset of Temporally Consistent Instance Annotations for 4D Plant Phenotyping

**DOI:** 10.1038/s41597-026-08326-5

**Published:** 2026-09-18

**Authors:** Jonas Bömer, Elias Marks, Facundo Ramón Ispizua Yamati, Cyrill Stachniss, Stefan Paulus, Anne-Katrin Mahlein

**Affiliations:** 1 Institute of Sugar Beet Research, Göttingen, Germany; 2 Center for Robotics, University of Bonn, Bonn, Germany

## Abstract

Spatio-temporal 4D plant phenotyping requires high-quality datasets with precise and temporally consistent annotations. However, such datasets are currently limited due to the substantial effort required for data acquisition and manual annotation. To address this limitation, we present Sugar4D, a publicly available 4D plant phenotyping dataset of sugar beet acquired using a terrestrial LiDAR scanner. The dataset comprises 768 point clouds from 48 individual plants representing twelve genotypes, recorded semiweekly across 16 consecutive time points during the growing season. All point clouds are provided with temporally consistent, pointwise instance annotations at the organ level, enabling the tracking of individual leaves over time. Sugar4D includes 6778 annotated leaves corresponding to 675 unique leaf instances. In addition to the annotated point cloud data, we provide 58 plant- and five leaf-related morphological parameters for each plant and leaf at each time point, validated using a 3D-printed plant reference model and invasive manual reference measurements. Sugar4D supports the development and evaluation of methods for plant instance segmentation, temporal registration, organ tracking, and spatio-temporal morphological analysis.

## Background & Summary

Understanding the temporal development of plant morphology is essential for advancing plant research, breeding, and practical agriculture. Spatio-temporal four-dimensional (4D) phenotyping approaches, which capture plant structure across multiple developmental stages, can provide critical insights into plant health and growth dynamics beyond those obtainable from static measurements^1,2^. Recent advances in sensor technology, computing hardware, and data-driven analysis methods have enabled rapid progress in three-dimensional (3D) plant phenotyping^3,4^, allowing the automated extraction of ordinary and complex structural traits such as plant height or leaf inclination angle from point cloud data. These traits provide important phenotypic information for comparing plant geno- and phenotypes^5^, potentially useful for analyzing plant stress responses and interactions with environmental factors.

At the same time, the emergence of high-throughput phenotyping platforms, ranging from stationary gantry-based systems to uncrewed aerial vehicles, has made it possible to repeatedly capture plants over time. Time series data is already being used to model disease outbreaks^6^ or predict future plant growth^7^. Within spatio-temporal 4D plant phenotyping, researchers analyze the dynamic alteration of 3D parameters, which facilitates a deeper understanding of both individual plant and plant population development. These insights provide previously inaccessible knowledge about the interaction of genes, phenotypes, and the environment, which is essential for understanding the formation mechanism of distinct biological traits^8^. However, research in this domain is still in its early stages.

Generating 4D data requires substantial time, cost, and labor. Consequently, only a limited number of spatio-temporal datasets are publicly available. Table 1 provides an overview of available datasets that comprise 3D point clouds of plants at multiple points in time.

**Table 1:** Overview of publicly available 4D plant phenotyping datasets, ordered by annotation type and publication date.

Name	Crop	Setting	Modality	Attributes	Number of plants	Number of genotypes	Temporal range/Time points	Annotation type	Time cons. labels
Khanna^31^	Sugar beet	Greenhouse	Passive stereo depth	x,y,z, r,g,b, spectral	180	1	10 weeks/16	✗	✗
Stuart^32^	Wheat	Greenhouse	3DGS, NeRF	x,y,z, r,g,b	20	6	15 weeks/6	✗	✗
Plant3D^33^	Tomato, Tobacco, Sorghum, Arabidopsis	Greenhouse	Laser triangulation	x,y,z	17, 8, 12, 63	1, 1, 1, 10	varying/varying	✗	✗
MuST-C^34^	Sugar beet, Potato, Maize, Wheat, Wheat+faba bean, Soybean	Field	SfM-MVS, Laser triangulation, LiDAR	x,y,z, r,g,b	n.a.	1, 2, 4, 1, 2, 2	16-23 weeks/ varying	✗	✗
Crops3D^35^	Tomato, Maize, Wheat, Potato Rice, Rapeseed, Cotton, Cabbage	Greenhouse, Field	LiDAR, SfM-MVS, Structured light	x,y,z, r,g,b	83, 225, 148, 118, 84, 150, 176, 196	n.a.	n.a./varying	semantic	✗
Galba^36^	Common bean, Cowpea, Lima bean, Mungbean	Outdoors (micro-plots)	Laser triangulation	x,y,z	n.a.	n.a.	varying/varying	semantic, instance (incomplete)	✗
Pheno4D^2^	Tomato, Maize	Greenhouse	Laser triangulation	x,y,z	7, 7	n.a.	1-3 weeks/20, 12	instance (incomplete)	*✓*
LAST-Straw^14^	Strawberry	Greenhouse	Structured light	x,y,z,r,g,b	6	2	11 weeks/14	instance (incomplete)	*✓*
Soybean-MVS^37^	Soybean	Greenhouse	MVS	x,y,z,r,g,b	10	5	16 weeks/13	instance	✗
Sugar4D^15^ (ours)	Sugar beet	Greenhouse	LiDAR	x,y,z,nx,ny,nz	48	12	8 Weeks/16	instance	*✓*

Our dataset is listed last for direct comparison (n.a. = not available).

These datasets vary considerably in multiple respects, starting with fundamental aspects like the measured crop, experimental settings or the technology used for 3D reconstruction. They also differ in aspects like individual plant or genotype count and their overall temporal range. All datasets lack some critical component necessary for broad application in 4D plant phenotyping.

One of the most significant factors that distinguishes the listed datasets is the availability of precise pointwise manual annotations of plant organs, usable for semantic and instance segmentation tasks. The benefit of raw unlabeled Cartesian point cloud data for phenotyping is limited, and reliable plant organ segmentation is essential for obtaining meaningful insights into plant morphology^9,10^. Although automatic plant segmentation keeps improving through the increasing use of deep learning, the creation of precisely annotated, high quality 4D datasets is essential^10^. However, manual pointwise annotations are a time- and labor-intensive task, a fact that is reflected in the limited number of datasets that include them. While some datasets are accompanied by semantic labels, instance labels are often found to be incomplete when they are provided (Table 1). This limitation constrains the development, comparison, and validation of plant instance segmentation, and subsequent analyses that depend on segmentation remain challenging to perform reliably. This underlines the significance and demand for publicly accessible instance-labeled datasets, whether for training and comparing plant instance segmentation networks or for establishing methods that analyze plant morphology based on the provided annotations.

Additionally, comprehensive 4D phenotyping requires not only precise, but also temporally consistent instance annotations. That is, the label of a particular leaf must remain identical across all consecutive time points^2^. This consistency is important for temporal point cloud registration, plant organ tracking, and subsequent growth analysis. Preliminary research on these subjects has been conducted^1,11– 13^, but their scope is limited by the availability of data, scalability, and consistency^13^. This highlights the need for publicly accessible, large-scale 4D datasets with consistent labeling becomes evident^1,11,12^. Such datasets facilitate the training, evaluation, and advancement of data-intensive methods in this domain. So far, only Pheno4D^2^ and LAST-Straw^14^ provide temporally consistent instance annotations, yet these are incomplete and do not cover the entire dataset. In addition to incomplete annotations, both datasets are limited to six or seven individual plants each, with LAST-Straw including only two genotypes, while Pheno4D does not provide any genotype information.

We address this gap by introducing Sugar4D^15^, an entirely temporally consistent labeled 4D dataset of sugar beet. Sugar beet is an economically important crop in temperate regions and a major target of breeding programs. Throughout its development, it continuously produces new leaves while older leaves undergo senescence, resulting in rich temporal growth dynamics and progressively increasing architectural complexity. Sugar4D^15^ encompasses point clouds collected using a high precision Light Detection and Ranging (LiDAR) sensor of twelve different sugar beet genotypes. Each genotype is represented in fourfold repetition, resulting in 48 individual plants with a wide range of morphological differences. The dataset has a high temporal resolution, with semiweekly measurements across a period of eight weeks, resulting in 16 consecutive time points. All 768 point clouds include temporally consistent pointwise instance labels, allowing analysis of temporal growth dynamics at the leaf scale. Sugar4D^15^ enables the training and evaluation of both temporally consistent and non-consistent plant instance segmentation networks. The substantial manual annotations provide a reliable baseline. Across all time points, the dataset contains 6778 annotated leaves, spread across 675 individual leaf instances. In addition to the point cloud data, we provide phenotypical data in form of 58 plant-related and five leaf-related morphological parameters, automatically extracted for each plant or leaf at each time point. Completing this data set, we provide validation data for both parameter types. While plant-related parameters can be referenced using a 3D-printed plant reference model^16^, leaf-related parameters are validated using invasive manual reference measurements of 450 individual leaves. Based on this data, Sugar4D^15^ supports the implementation and validation of common and novel spatial and spatio-temporal morphological parameters. So far, we have used the dataset to analyze the benefit of spatio-temporal data for morphology-based plant genotype differentiation^5^. Other possible use cases are 4D plant growth modeling and prediction^17,18^, temporal point cloud registration and organ tracking^1,11–13^, or the generation of synthetic plant point cloud data^19–21^.

## Methods

The data collection and processing protocols align with the procedures outlined in Bömer *et al*.^5^, although we did a final revision of the manual labels before the publication of the dataset. The methods employed to generate Sugar4D^15^ are described in detail below.

### Experimental Design

**Fig. 1:**
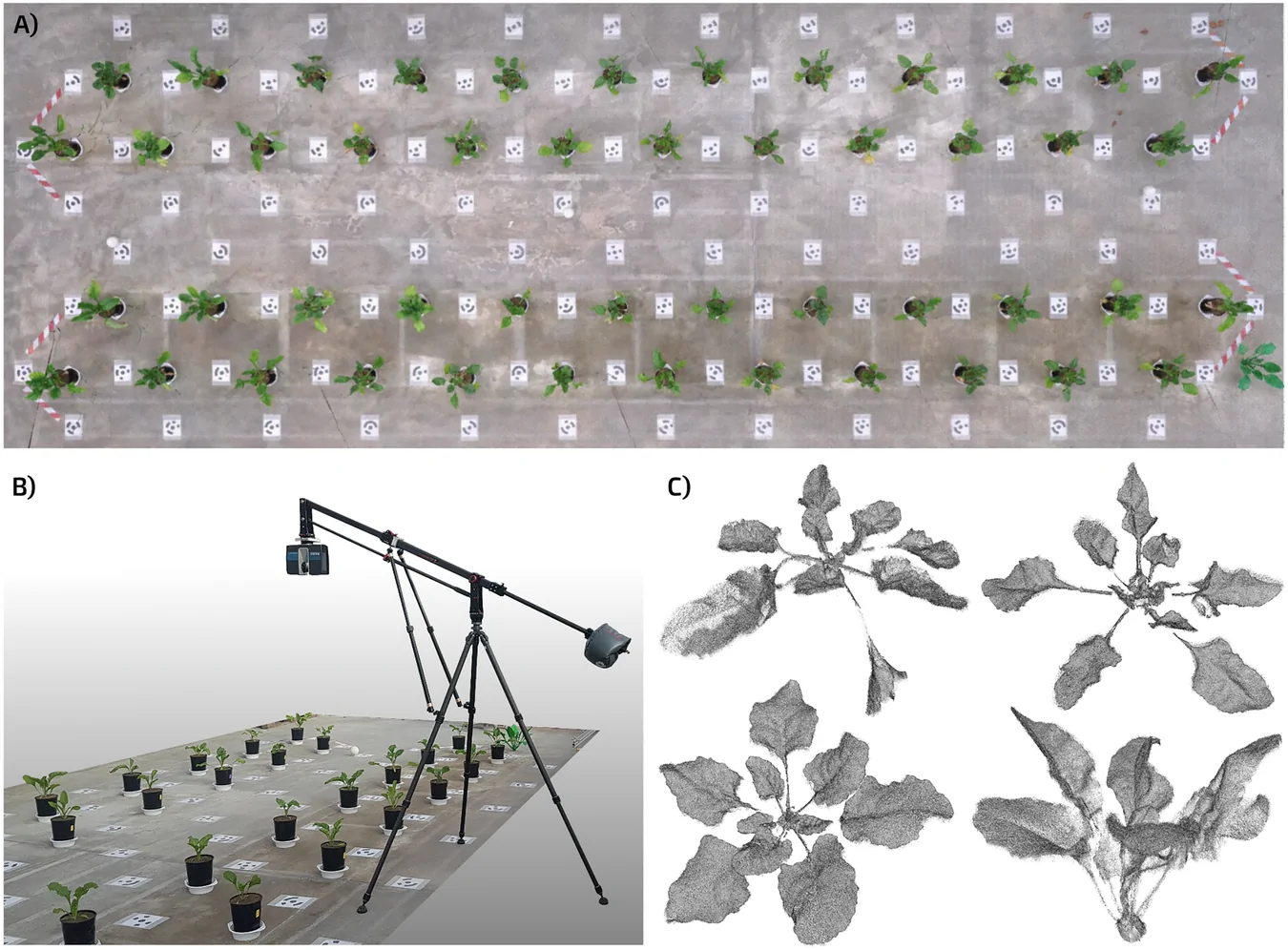
Experimental setup and data acquisition conditions of Sugar4D. Overview of the conducted greenhouse experiment at 72 days after sowing, including the utilized 3D-printed reference plant (bottom right) (**A**). We collected the dataset using a terrestrial LiDAR sensor, suspended upside-down on an extension arm mounted to a tripod (**B**). The final dataset encompasses preprocessed point clouds of sugar beet plants. Four different genotypes are depicted (from top left to bottom right: 9, 11, 3, and 4), highlighting the diversity of morphological traits present in the dataset (**C**).

**Fig. 2:**
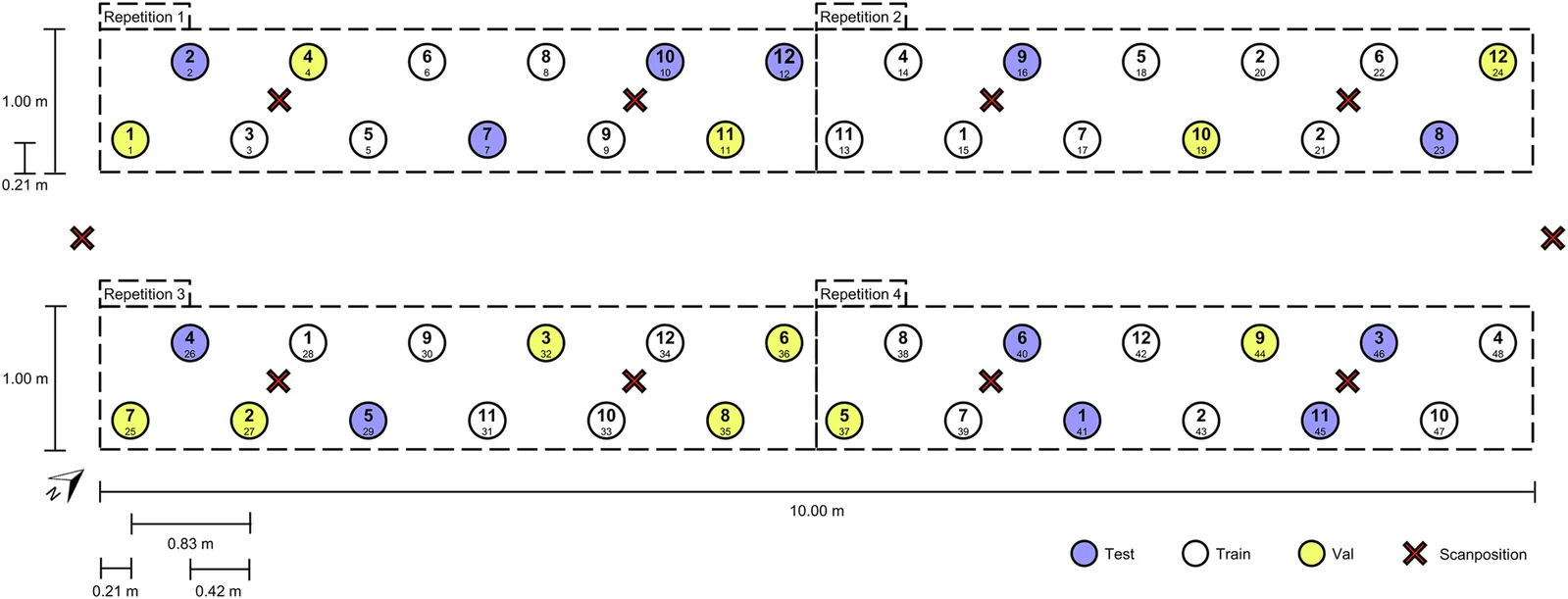
Experimental layout of the greenhouse trial. Each plant position is labeled with the genotype identifier (large number) and the global flowerpot number (small number). Terrestrial scanning positions are indicated by cross markers. The affiliation of each plant to data partitions for machine learning is color-coded.

We carried out the experiment under controlled greenhouse conditions at the Institute of Sugar Beet Research in Göttingen, Germany, between September and December 2021. Twelve different sugar beet (*Beta vulgaris*) genotypes were cultivated. These comprised eight non-commercial breeding lines (genotypes 1-8) exhibiting pronounced morphological variation, and four commercially available varieties (genotypes 9-12), which display more subtle phenotypic differences but are of higher agronomic relevance. We designed this selection to encompass a wide range of morphological growth variability in the dataset while retaining patterns representative of practical agricultural conditions. Examples of the resulting structural diversity are illustrated in Fig. 1C. We cultivated all genotypes in fourfold repetition, resulting in 48 plants in total. The experiment layout followed a fully randomized block design. To avoid interactions among plants, such as shading or physical contact, we spatially separated the flowerpots. The experiment layout is portrayed in detail in Fig. 2. Figure 1A shows an overview of the experimental setup, illustrating the spatial layout and growing progress at 72 days after sowing (DAS).

We used an automated climate control system to maintain a constant greenhouse temperature of 20^∘^C in the greenhouse (20.1  ± 4.9 ^∘^C, 41.2  ± 12.5% relative humidity), while artificial illumination with sodium-vapor lamps provided a 16-hour daily photoperiod. For the germination process, we placed the seeds in moist filter paper for four days. We transferred four seeds of the same genotype to a single two-liter flowerpot filled with a pre-fertilized substrate composed of equal parts sand and topsoil by weight (Gustav Lehmann Mörtel- u. Kieswerke GmbH, Germany). The selected flowerpot volume was adequate to rule out the potential impact of root or shoot growth restriction on plant morphology for the duration of the experiment. By 26 DAS, the plants in the flowerpots were gradually thinned, and only the most advanced plant was retained for the experiment.

We maintained all plants under a uniform management protocol, including regular monitoring, integrated pest management using systemic fungicide (Tachigaren 70 WP; Mitsui Chemicals Crop & Life Solutions, Japan), beneficial organisms (*Steinernema feltiae*, *Stratiolaelaps scimitus*; Katz Biotech AG, Germany), and fertilizer application (liquid universal fertilizer (4% nitrogen, 2% phosphorus pentoxide, 2% potassium oxide), 50 mL, 1:40 ratio) at 81 and 88 DAS to ensure comparable growth conditions and plant health across all genotypes and replicates. Irrigation was conducted manually as required, as well as on the evenings prior to the next day’s data collection to ensure a sufficient turgor.

### 3D Data Acquisition

We consistently performed data acquisition in the early morning hours to ensure maximal leaf turgor and minimize diurnal variation in leaf attitude. The 3D point cloud data was acquired using a high precision terrestrial LiDAR sensor (Faro Focus S70; Faro Technologies Inc, USA), which operates at a wavelength of 1550 nm. The sensor measures up to 1 million points per second in a range of 0.6 m to 70.0 m with a ranging error of  ± 1.0 mm. For our experiment, we set the scanner resolution to 3.1 mm at a distance of 10.0 m to balance point density and scanning speed.

We suspended the sensor in an inverted orientation on a camera arm (Mini Crane M1-III; iFootage, USA) attached to a tripod, as depicted in Fig. 1B. This measuring setup enabled direct positioning of the sensor above the plants, thereby improving visibility of inner plant structures and reducing occlusion effects compared to common upright terrestrial laser scanning configurations. The camera arm was supported by lateral stabilizers to prevent any vibrations of the system introduced by the rotary motion of the LiDAR scanner.

To ensure high spatial resolution and low occlusion, we collected data from ten scanning positions arranged in a circular pattern. The individual scanning positions are depicted in Fig. 2. The complete data acquisition session required approximately one hour. To enable accurate registration of the individual scans into a unified 3D point cloud, we strategically positioned nine spherical reference targets (Ø145 mm; Laserscanning Europe GmbH, Germany) in and around the experiment.

We conducted measurements at 16 consecutive time points at intervals of three to four days, with one exception between time points nine and ten, where the interval extended to seven days due to a sensor malfunction. The measurements started 37 DAS when plants had reached BBCH growth stage 12 to 14 and lasted 56 days until 93 DAS. This resulted in a total number of 768 point clouds.

### Point Cloud Processing

After data collection in the greenhouse, we processed each individual scan using laser scanning software (Faro Scene; Faro Technologies Inc, USA). The processing included multiple filtering steps (stray point, low-reflectivity, edge artifact), manual annotation of the registration targets, and final registration into a unified point cloud with sub-millimeter point density utilizing the annotated targets. We monitored registration accuracy for each time point (see section Technical Validation).

After registration, we transformed point clouds from all 16 time points into a standardized Cartesian coordinate system using three floor-mounted photogrammetric targets. Individual plant point clouds were automatically extracted based on their known coordinates. The ground plane was removed using Random Sample Consensus^22^, followed by removal of remaining outliers were removed using a statistical outlier filter^23^.

### Point Cloud Annotation

We annotated the point clouds using an online data labeling platform (https://segments.ai; Segments.ai BV, Belgium). A single annotator performed the initial annotations to ensure label consistency, and an independent second annotator revised them to enhance overall label accuracy. An independent third annotator reviewed and did a final correction of all annotations to ensure label integrity. The annotation time varied with plant size and time point, ranging from a few minutes for emerging plants to multiple hours for fully grown plants. In total, the annotation of the dataset took several months to complete.

**Fig. 3:**
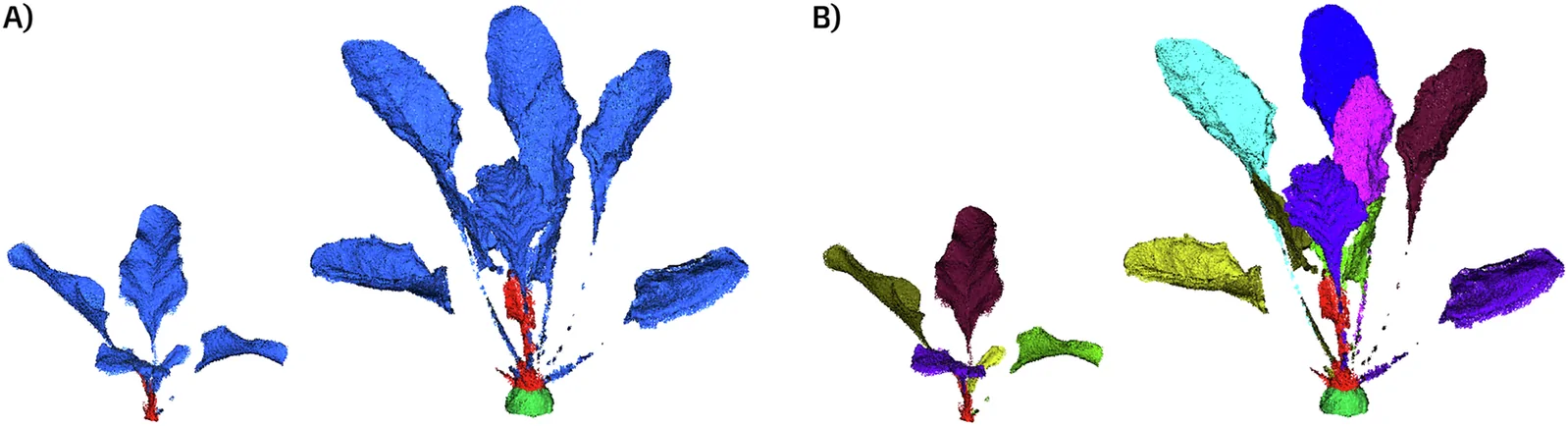
Available pointwise annotations of Sugar4D, showing semantic labels for class leaf (blue), core (red), and taproot (green) (**A**), and corresponding instance labels for class leaf (**B**).

**Fig. 4:**
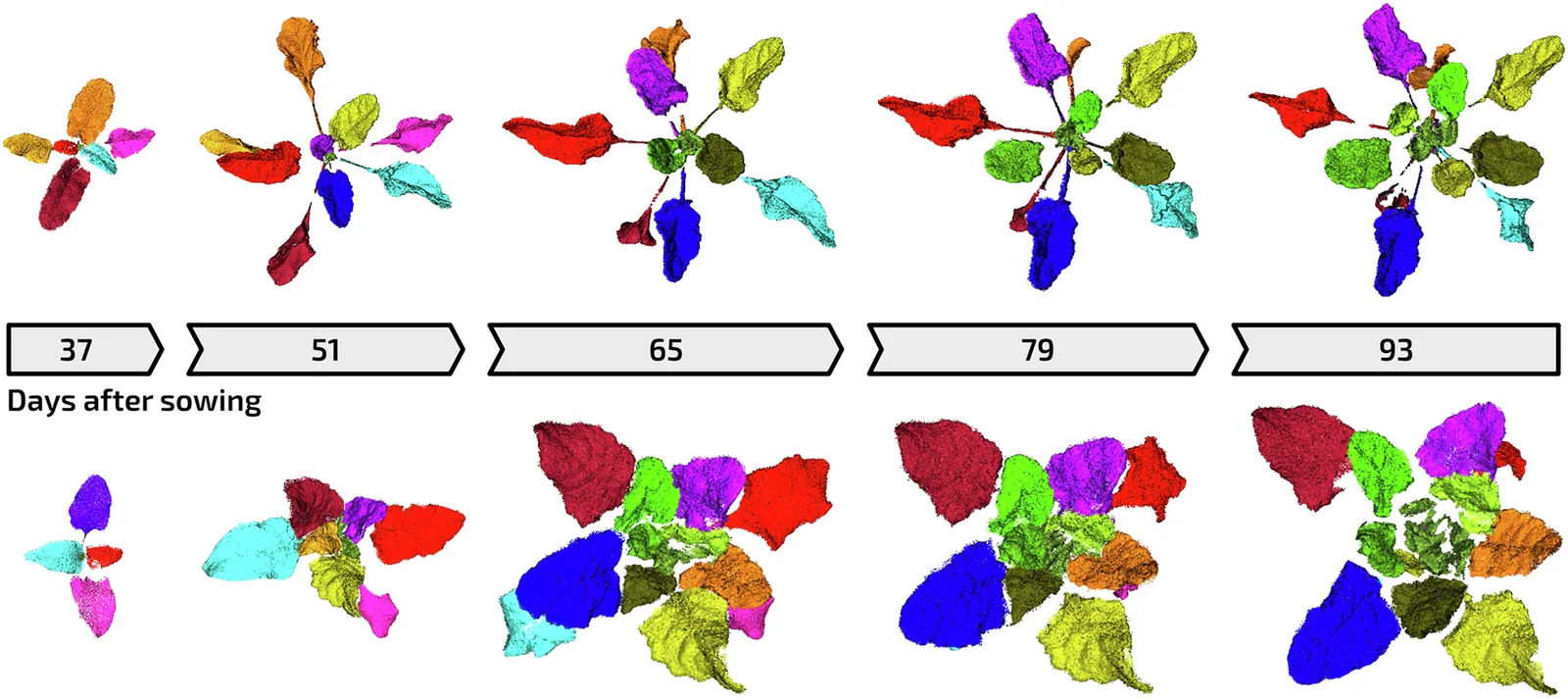
Temporally consistent annotated point clouds of Sugar4D, illustrating temporal leaf development and architectural differences between growth types covering five time points (37, 51, 65, 79, and 93 days after sowing). The upper row shows genotype 9 with a sparse growth type and loosely arranged leaves, while the lower row shows genotype 8 with a compact growth type and densely packed leaves.

Before semantic labeling, we manually refined the point clouds by removing the flowerpot, soil, and remaining outliers. The cleaned plant point cloud was then segmented into the three semantic classes of leaf, core, and taproot, as depicted in Fig. 3A. core signifies the innermost plant region at the center of the leaf rosette, where point density and acquisition precision of the LiDAR scanner proved insufficient for reliable manual labeling of emerging leaves. Refinement of the semantic leaf annotations led to the generation of instance labels for each leaf, as depicted in Fig. 3B. We generated the instance labels of Sugar4D^15^ to maintain temporal consistency, which enables the monitoring of individual leaves throughout their respective growth duration. Figure 4 illustrates a representation of these consistent instance labels for two plants of different growth types.

### Morphological Parameters

Besides extensive annotations, Sugar4D^15^ provides automatically extracted single plant-related and single leaf-related morphological parameters. A total of 58 single plant-related parameters are provided for all 768 plant point clouds. They encompass 37 plant-based and 21 leaf-based parameters. The extraction pipeline was implemented in Python^24^ (v.3.10.0) and relies primarily on NumPy^25^ (v.1.26.4), SciPy^26^ (v.1.14.0), and Open3D^23^ (v.0.18.0) for point cloud processing. It is described in detail in Bömer *et al*.^5^, where Table 1 and Table 2 provide the formal definition of each released plant- and leaf-related parameter, the complete list of dependencies and versions is stated, and the corresponding Python code is published as supplementary material (see section Code availability). The parameters include standard traits such as plant height and width, as well as more complex ones such as Leaf Overlap Index and Gap Fraction. Several parameters require a defined plant growth point, which we manually annotated in the initial recording and propagated to all subsequent time points, as the flowerpot positions remained fixed throughout the experiment. We used a 3D-printed reference model of a sugar beet plant following Bömer *et al*.^16^ to provide a method to validate the extracted parameters, since manual reference measurements cannot be conducted for most higher dimensional parameters. The model was included in the data acquisition process 15 times (see Fig. 1A).

In addition to single plant-related parameters, we provide five single leaf-related parameters for all 6778 annotated leaves of the dataset. These were automatically extracted using a template matching approach based on Marks *et al*.^27^ and encompass the Leaf Length, Blade Length and Blade Width, Petiole Length, and Leaf Area. We performed invasive measurements after the final data acquisition at 93 DAS to provide manual reference data for four leaf-related parameters and validate the template matching approach. Therefore, we detached leaves from the taproot and measured the Leaf Length, Blade Length and Blade Width, and Petiole Length of 450 leaves according to official variety testing protocols^28^. Leaf Area was not measured manually because no reliable invasive manual reference measurement can be conducted given the irregular curvature and the blistered geometry of sugar beet leaves. All measurements are provided together with their corresponding leaf instance label.

## Data Records

**Fig. 5:**
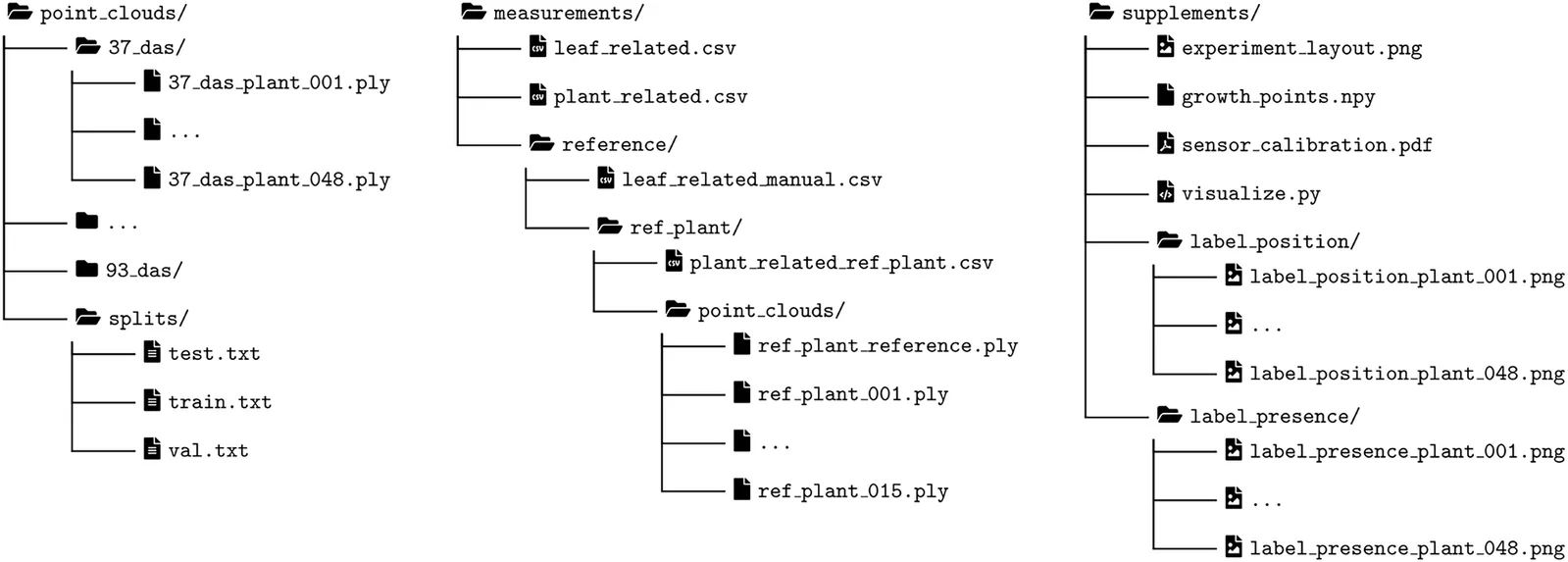
Directory structure of Sugar4D. The dataset is organized into three main sections: point_clouds contains the labeled plant point cloud data and predefined data splits, measurements includes the automatically extracted morphological parameters along with corresponding reference measurements and 3D models of the utilized reference plant, and supplements provides additional experiment related information, a Python script for visualization of the dataset, and technical validation results.

Sugar4D^15^ is available at the public access repository bonndata under CC BY 4.0 license. The dataset has a total size of 5.8 GB. Figure 5 depicts the repository structure, which is split into three main directories. These comprise the annotated point cloud data, automated morphological measurements together with automatic and manual reference measurements, and additional supplementary material including the technical validation results.

The main directory point_clouds contains 17 subdirectories, 16 of which contain point cloud data. Each recording subdirectory is named after the recording time point encoded in DAS and contains 48 individual point clouds. Users can attribute the nomenclature of the plant point clouds to individual genotypes using the experiment layout plan (Fig. 2). The point clouds are stored in a binary *.ply* file format^29^, which supports user-defined elements and properties, such as label indices, beyond the standard point cloud representation. Each vertex in the point cloud contains the following information:

- coordinates  → *x*, *y*, *z*
- normals  → *nx*, *ny*, *nz*
- annotations  → *label_semantic*, *label_instance*

The annotations are provided in form of integer values:

- taproot  → semantic: 0; instance: -2
- core  → semantic: 1; instance: -1
- leaf  → semantic: 2; instance: 0-19

The remaining subdirectory splits provides a predefined dataset partition intended for machine learning applications. We performed partitioning at the plant level and used a random 50:25:25 split for training, testing, and validation, while ensuring that each cultivated genotype is represented in all three subsets.

The main directory measurements contains automatically extracted single plant- and leaf-related morphological parameters for all point clouds (plant_related.csv, leaf_related.csv). It includes the subdirectory reference, which provides all data required for parameter validation. This subdirectory contains manual measurements of 450 individual leaves (leaf_related_manual.csv), validation results for all extracted parameters (plant_related_ref_plant.csv), and the corresponding point clouds needed for validation (ref_plant/point_clouds).

The main directory supplements contains additional resources that support the use of the dataset. It includes the experimental layout (experiment_layout.png) and the calibration certificate of the employed LiDAR sensor (sensor_calibration.pdf). The directory also contains the growth point of each plant, which is required for computing, validating, and extending derived parameters (growth_points.npy). For a straightforward visual inspection of Sugar4D^15^, we provide a basic Python script (visualize.py). It requires Open3D^23^ (version ≥ 0.16.0) and allows the visualization of individual plants with semantic or instance annotations at a single time point, over a specified time period, or across the full dataset. The script can be executed from the command line as follows:


python visualize.py --plant PLANT_ID --label LABEL_TYPE --time_point TIME_POINT


where:

- PLANT_ID: plant identifier, e.g. 001
- LABEL_TYPE: label type, either semantic or instance
- TIME_POINT: time selection: a single index (e.g. 1), a time period (e.g. 1-9), or all

Annotation accuracy is assessed for each plant in label_position and label_presence. Additional details are provided in the section Technical Validation.

## Technical Validation

### Spatial Point Cloud Integrity

The functionality and precision of the utilized LiDAR sensor is documented by the enclosed sensor calibration certificate. It verifies the sensor’s high 3D position accuracy and low ranging noise, with errors  <1.0 mm.

We collected ten individual scans from varying positions within the experimental setting and registered them into a unified point cloud. To quantify the spatial integrity of the resulting point clouds, we evaluated registration quality using target- and scan point-based statistics exported from the registration software. While the target-based statistics provide registration quality information of the scene based on the utilized spherical reference targets, the scan point statistics evaluate the agreement between the overlapping parts of two scans. These metrics assess whether scan alignment is both geometrically consistent and supported by dense surface agreement. Summary statistics per time point are reported in Table 2.

**Table 2:** Registration quality metrics for individual LiDAR scans at each time point, including target-based distance/horizontal/vertical errors and scan-based point errors and overlap (n=10).

Days after sowing	Target statistics						Scan point statistics		
	Mean distance error [mm]	Max. distance error [mm]	Mean horizontal error [mm]	Max. horizontal error [mm]	Mean vertical error [mm]	Max. vertical error [mm]	Mean point error [mm]	Max. point error [mm]	Minimum overlap [%]
37	3.3	8.5	1.7	5.0	2.5	8.5	2.4	3.7	82.3
40	3.3	10.9	1.4	5.1	2.7	10.9	2.0	3.5	84.6
44	3.0	9.6	1.3	3.7	2.4	9.6	1.9	3.0	84.4
47	2.7	9.2	1.7	7.4	1.8	8.8	1.9	2.6	82.4
51	3.3	9.8	1.5	4.6	2.5	9.7	1.7	3.7	85.5
54	2.7	9.4	1.4	4.2	2.0	9.1	1.9	2.7	83.9
58	2.5	8.9	1.6	4.9	1.6	8.7	2.0	2.6	81.2
61	2.3	7.4	1.5	4.5	1.5	7.0	1.9	2.4	82.8
65	2.2	6.4	1.5	4.1	1.3	6.4	2.1	2.7	83.2
72	2.6	8.3	1.4	4.0	1.9	8.2	2.0	2.6	84.2
75	2.5	7.6	1.5	4.4	1.6	7.5	1.9	2.5	84.0
79	2.6	7.8	1.5	4.5	1.8	7.6	2.1	2.5	81.1
82	2.9	9.2	1.7	6.3	2.0	9.2	2.0	2.9	79.7
86	2.1	5.3	1.3	3.9	1.3	4.5	0.9	1.2	98.4
89	2.3	7.6	1.4	4.3	1.6	7.1	1.1	2.1	97.0
93	1.8	7.4	0.7	2.1	1.5	7.2	0.9	1.1	73.4
Mean	2.6	8.3	1.4	4.6	1.9	8.1	1.8	2.6	84.3
SD	0.4	1.4	0.2	1.1	0.4	1.5	0.4	0.7	6.0

The target-based statistics show consistently low errors. Across all time points, the mean target distance error averages 2.6  ± 0.4 mm, with mean horizontal and vertical distance errors of 1.4  ± 0.2 mm and 1.9  ± 0.4 mm, respectively. Maximum target distance errors remain low overall, with mean maxima of 8.3  ± 1.4 mm distance, 4.6  ± 1.1 mm horizontal, and 8.1  ± 1.5 mm vertical error. These results indicate the absence of systematically incorrect registrations or isolated mismatches.

The scan point statistics confirm these findings. The mean point error across overlapping regions is 1.8  ± 0.4 mm across all time points, with a mean maximum point error of 2.6  ± 0.7 mm. Due to the ten individual 3D scans per time point, the minimal overlap of all individual scans is very high with 84.3  ± 6.0%.

Overall, both target- and scan point-based measures indicate stable, millimeter-level registration accuracy across all time points, supporting high spatial consistency of Sugar4D^15^.

### Temporal Annotation Integrity

The spatial precision of the manual annotations is ensured by our labor-intensive, consecutive three-step annotation procedure. Given the dataset’s size, occasional temporal instance annotation errors may occur and can bias downstream analyses. To ensure label integrity, we verify the temporal consistency of semantic and instance annotations and track label positions over time to detect temporal label permutations.

**Fig. 6:**
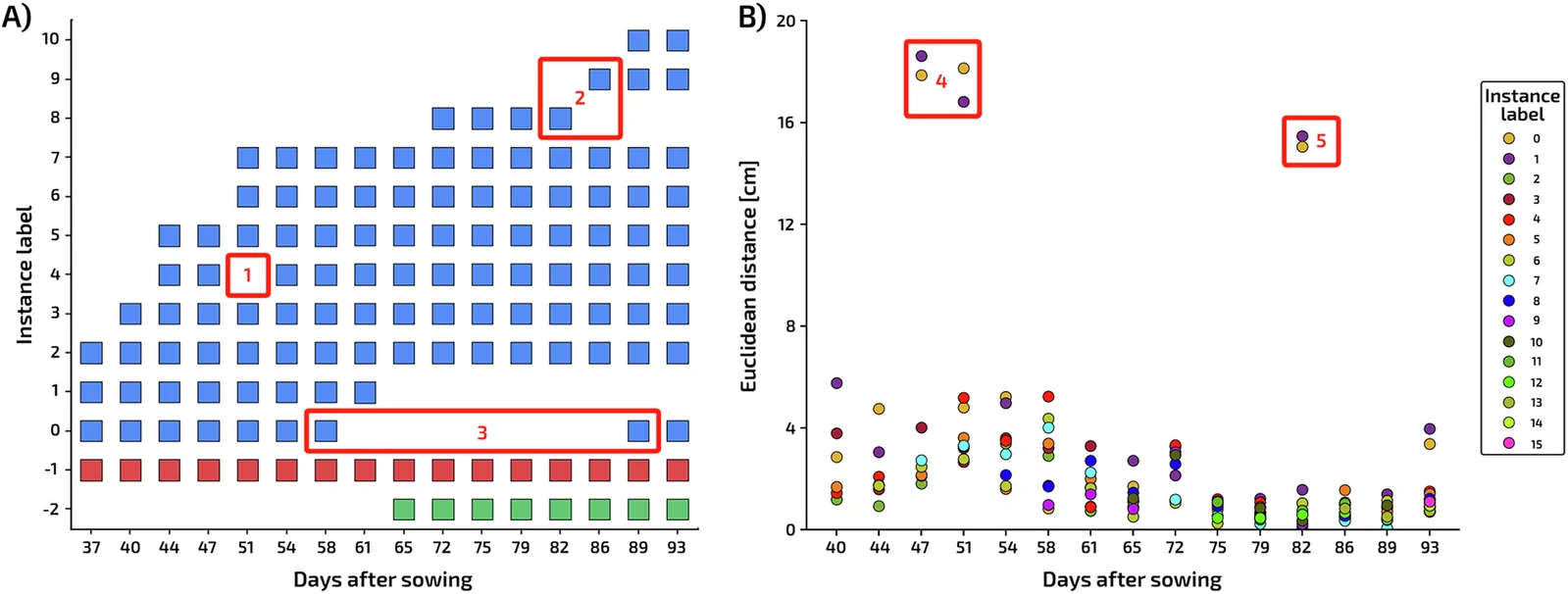
Visualization of the annotation integrity diagrams, highlighting common temporal annotation errors (**A**) alongside within-plant label permutations (**B**). Detectable errors are marked with red squares. (**A**) shows the temporal label occurrence per plant, where each square represents the presence of a label. Detected errors include single-frame mistaken identity (1), persistent instance label shifts (2), and label re-use (3). (**B**) visualizes within-plant temporal label center translation. Detected errors include single-frame (4) and constant permutations (5).

To assess temporal consistency and identify potential temporal annotation errors, we extracted all labels for each plant at each time point to obtain a per-plant temporal annotation overview diagram. Figure 6A shows an example of temporally inconsistent annotations. This visualization enabled the identification and correction of three temporal annotation error types: single-frame omissions visible as isolated missing labels (Fig. 6A1), persistent label permutations reflected by consecutive label disappearance and reappearance (Fig. 6A2), and temporal label reuse indicated by the delayed reintroduction of a previously used instance label (Fig. 6A3).

To prevent accidental within-plant label permutations, we quantified the positional change of each label by computing the translation of its centroid relative to the previous time point. Permutations appear as sudden increases in centroid displacement of two instance labels, as shown in Fig. 6B. Single-frame permutations produce two consecutive high displacement values due to the bidirectional label interchange (Fig. 6B4), whereas temporally constant permutations yield a single sustained increase due to a one-time label change (Fig. 6B5). A sudden increase in displacement provides a strong indication that a label permutation is present, although it may also result from differential growth or turgor changes.

To quantify the temporal consistency of leaf instance identity in the released annotations, we summarized the centroid displacement of all leaf instances between consecutive time points across all 48 plants. Table 3 reports the resulting distribution per time interval. The displacement decreases over the course of the experiment, from a mean of 2.85 cm in the first interval to below 1.00 cm in the later intervals, which is consistent with rapid leaf growth in early developmental stages and a stabilization of leaf position as the plants mature. The 95^th^ percentile remains close to the mean across all intervals, indicating that the large majority of displacements correspond to genuine growth and turgor variation. The few larger values per interval, reflected by the difference between the 95^th^ percentile and the maximum, were individually inspected as part of the three-step annotation procedure to identify label permutations.

**Table 3:** Leaf instance centroid displacement between consecutive time points across all 48 plants.

Interval [DAS]	Mean [cm]	Median [cm]	SD [cm]	P95 [cm]	Max [cm]	Number of leaves
37-40	2.85	2.52	1.72	5.96	11.53	198
40-44	2.88	2.49	1.72	6.09	13.34	259
44-47	2.84	2.77	1.24	4.90	8.20	292
47-51	2.91	2.58	1.80	5.71	16.33	345
51-54	2.41	2.11	1.48	5.21	10.06	382
54-58	2.65	2.14	1.74	6.39	9.05	400
58-61	1.63	1.33	1.07	3.69	7.87	413
61-65	1.62	1.28	1.16	3.79	7.72	419
65-72	1.95	1.68	1.23	4.18	11.42	435
72-75	0.95	0.62	1.11	2.94	12.78	466
75-79	0.76	0.58	0.67	1.76	7.47	471
79-82	0.90	0.64	0.82	2.43	8.00	477
82-86	1.03	0.83	0.78	2.19	6.31	497
86-89	0.94	0.71	0.89	2.54	10.18	514
89-93	1.49	1.03	1.75	3.62	17.42	535

Displacement is reported for leaf instances only, excluding taproot and core. Intervals are labeled by their days after sowing (DAS) transition.

We reviewed all annotated instances and corrected them as needed, so that the released dataset contains no temporal label permutations. Our analysis does not quantify point-level annotation accuracy or independent annotator agreement, in consequence of our consecutive labeling approach. The final temporal annotation examinations for each plant are provided in Sugar4D^15^.

### Morphological Parameter Validation

The 58 released plant-related parameters of Sugar4D^15^ were validated against a 3D-printed sugar beet reference model following Bömer *et al*.^16^. Repeated scanning of this reference model under varying conditions allows the extracted parameters to be compared against benchmark values derived from the corresponding digital base model, providing an assessment of their accuracy and repeatability. Based on 15 repeated measurements, we quantified the systematic bias and the scan-to-scan variability of the extracted parameters. Table 4 reports the resulting statistical analysis for all plant-related parameters, summarizing complementary metrics that distinguish accuracy-related bias from precision-related scatter.

**Table 4:** Relative error metrics for all morphological plant-related parameters derived from all scans of the 3D-printed plant reference model compared to the digital base model (n = 15).

Parameter	MPE [%]	MAPE [%]	RMSPE [%]	MaxAPE [%]	SD [%]	CV [%]
Blade Length IQR	−31.28	31.28	32.69	49.81	9.52	13.86
Blade Length Maximum	−12.97	12.97	13.11	16.00	1.94	2.23
Blade Length Mean	−6.85	6.85	7.07	10.20	1.72	1.84
Blade Length SD	−21.58	21.58	21.81	27.13	3.16	4.03
Blade Width IQR	36.64	36.64	40.09	79.14	17.20	12.59
Blade Width Maximum	76.70	76.70	88.21	195.75	43.58	24.66
Blade Width Mean	44.75	44.75	45.57	66.11	9.02	6.23
Blade Width SD	39.37	39.92	55.13	182.17	38.61	27.70
Convex Hull Compactness	−0.22	0.49	0.72	2.08	0.58	0.59
Convex Hull Perimeter	−1.16	1.16	1.19	1.58	0.25	0.25
Convex Hull Surface Area	−3.25	3.25	3.40	4.72	0.99	1.03
Convex Hull Volume	−4.95	4.95	5.17	7.20	1.50	1.58
Gap Fraction	−2.30	2.30	2.36	3.14	0.54	0.55
Gap Fraction 3D	6.57	6.57	6.68	8.93	1.29	1.21
Ground Cover Area	−11.52	11.52	12.12	20.32	3.79	4.28
Height IQR	−6.12	6.65	7.73	18.18	3.96	4.22
Height Maximum	−2.01	2.01	2.09	2.86	0.59	0.60
Height Mean	−1.14	2.18	2.77	7.04	2.53	2.56
Height SD	−6.15	6.15	6.62	11.37	2.45	2.62
Height-Radius Index	−1.91	2.08	2.62	6.69	1.60	1.63
Height-Radius-Mean Index	−9.13	9.13	9.73	17.19	3.36	3.70
Height-Width Index	−0.44	1.04	1.35	3.54	1.28	1.29
Leaf Area IQR	−11.70	11.70	13.54	31.15	6.87	7.78
Leaf Area Maximum	−15.01	15.01	15.21	19.13	2.45	2.88
Leaf Area Mean	−11.02	11.02	11.34	16.24	2.64	2.97
Leaf Area SD	−16.32	16.32	16.41	19.52	1.74	2.08
Leaf Area Sum	−9.99	10.98	12.25	25.99	5.42	6.02
Leaf Length IQR	−13.28	13.28	15.41	34.86	7.82	9.01
Leaf Length Maximum	7.41	7.41	7.45	8.75	0.77	0.72
Leaf Length Mean	10.31	10.31	10.33	11.31	0.61	0.55
Leaf Length SD	−7.04	7.04	7.33	11.34	2.04	2.19
Leaf Overlap Index	7.66	7.66	7.74	9.88	1.12	1.04
Leaf Quantity	0.00	0.00	0.00	0.00	0.00	0.00
Leaf Spacing	−17.80	17.80	18.20	24.44	3.81	4.63
Normal Angle IQR	11.08	11.08	12.13	25.56	5.07	4.57
Normal Angle Kurtosis	81.86	81.86	85.16	123.55	23.48	12.91
Normal Angle Mean	7.72	7.72	8.08	15.55	2.40	2.22
Normal Angle SD	2.40	2.97	3.84	11.58	3.00	2.93
Normal Angle Skewness	−21.62	21.62	23.24	43.68	8.51	10.85
Points Quantity	−18.00	18.00	18.79	30.33	5.57	6.79
Projected Leaf Area Mean	−13.54	13.54	14.14	21.49	4.08	4.72
Projected Leaf Area	−13.54	13.54	14.14	21.49	4.08	4.72
Radius IQR	−2.79	2.98	3.57	9.25	2.24	2.31
Radius Maximum	0.34	0.76	0.96	2.80	0.90	0.90
Radius Mean	8.87	8.87	9.35	15.96	3.00	2.76
Radius SD	−0.45	1.53	2.22	6.56	2.25	2.26
Roundness	2.41	2.79	3.36	8.80	2.38	2.33
Spatial Extent IQR	−19.82	19.82	20.07	24.75	3.13	3.91
Spatial Extent Maximum	−0.88	1.02	1.20	3.12	0.81	0.82
Spatial Extent Mean	3.40	3.40	3.80	6.58	1.75	1.70
Spatial Extent SD	−10.93	10.93	11.55	18.85	3.73	4.19
Stem Length IQR	−18.12	18.12	20.23	43.54	8.99	10.97
Stem Length Maximum	29.08	29.08	29.23	33.09	2.97	2.30
Stem Length Mean	27.51	27.51	27.54	30.18	1.26	0.99
Stem Length SD	2.42	2.98	3.85	11.72	2.78	2.71
Width Aspect Ratio	8.96	9.01	10.79	26.26	6.06	5.56
Width Major Axis	−1.14	1.14	1.18	1.72	0.31	0.31
Width Minor Axis	7.69	8.03	9.59	26.57	5.86	5.44

The Mean Percentage Error (MPE) quantifies systematic offsets, the Mean Absolute Percent age Error (MAPE) and Root Mean Square Percentage Error (RMSPE) characterize the typical magnitude of deviations, the Maximum Absolute Percentage Error (MaxAPE) captures worst-case behavior, and the Standard Deviation (SD) and Coefficient of Variation (CV) describe variability. The majority of parameters are extracted reliably, with simple geometric traits such as Width Major Axis, Convex Hull Perimeter, Height Maximum, and Spatial Extent Maximum showing MAPE values below 2% and CV values below 1%. Leaf Quantity is reproduced exactly across all scans, as it is based on the manual annotations. In contrast, parameters derived from the leaf blade are the least reliable, with Blade Width Maximum and Normal Angle Kurtosis reaching MAPE values above 75%. The benefit of reporting both bias and scatter is illustrated by Stem Length Mean, which exhibits a high systematic deviation with an MAPE of 27.51% but a very low CV of 0.99%, indicating a stable yet systematically biased extraction. In general, the deviation increases with the complexity of the parameter calculation, and parameters based on the interquartile range or standard deviation are more sensitive than parameters based on maxima or means. Rather than omitting less reliable parameters, Table 4 documents the deviations of the complete released parameter set, enabling users to judge the suitability of each parameter for their specific application.

The released leaf-related parameters were validated against manual reference data measured on a single leaf basis. Contrary to the established BonnBeetClouds3D^30^ dataset, which provides average values per experiment plot, the manual reference data is provided per individual leaf. Table 5 reports the validation of our leaf parameter extraction algorithm based on template matching^27^ against this reference. Since no reliable manual reference data was available for Leaf Area, this parameter was excluded from validation.

**Table 5:** Error metrics describing the accuracy and agreement of our leaf template matching approach for single leaf-related parameter estimation, covering four of five released parameters for which manual reference measurements were feasible (n = 450).

Parameter	ME [cm]	MAE [cm]	MAPE [%]	RMSE [cm]	CCC
Leaf Length [cm]	1.63	1.92	11.31	2.30	0.92
Blade Length [cm]	1.63	1.87	17.22	2.24	0.83
Petiole Length [cm]	−2.35	2.38	28.01	2.69	0.56
Blade Width [cm]	0.25	1.09	15.40	2.61	0.47

The metrics characterize the systematic bias using Mean Error (ME), typical absolute and relative errors using Mean Absolute Error (MAE) and MAPE, the sensitivity to extreme deviations using Root Mean Square Error (RMSE), and the overall agreement using the Concordance Correlation Coefficient (CCC). Across parameters, MAE values between 1.09 cm and 2.38 cm indicate that most measurements are close to the manual reference, whereas consistently higher RMSE values indicate occasional large deviations. Blade Width shows low systematic bias but a substantially higher RMSE together with a reduced CCC, which indicates a small number of extreme outliers. Positive ME values for Blade Length and Leaf Length indicate a systematic overestimation, while the negative value for Petiole Length indicates a systematic underestimation.

Together, the validation against the 3D-printed reference model and the manual measurements documents the reliability of the released morphological parameters and supports their verification and interpretation in downstream analyses. The reference data can also be used to validate novel morphological parameters implemented on Sugar4D^15^ in the future.

## Data Availability

Sugar4D is available at the public access repository bonndata at https://doi.org/10.60507/FK2/IS8YBZ under CC BY 4.0 license.

## Code availability

A Python implementation for loading and visualizing the point clouds of Sugar4D^15^ is included in the dataset repository. The scripts used to extract the released plant- and leaf-related morphological parameters are published as supplementary material to Bömer *et al*.^5^ and are available at https://static-content.springer.com/esm/art%3A10.1007%2Fs11119-026-10337-6/MediaObjects/11119_2026_10337_MOESM2_ESM.zip.
